# Mechanisms and Active Compounds Polysaccharides and Bibenzyls of Medicinal Dendrobiums for Diabetes Management

**DOI:** 10.3389/fnut.2021.811870

**Published:** 2022-01-28

**Authors:** Mingjian Li, I. Gusti Surya Chandra Trapika, Suet Yee Sara Tang, Jun-Lae Cho, Yanfei Qi, Chun Guang Li, Yujuan Li, Meicun Yao, Depo Yang, Bowen Liu, Rong Li, Ping Yang, Guoyi Ma, Ping Ren, Xi Huang, Deshan Xie, Shaochao Chen, Min Li, Lan Yang, Ping Leng, Yong Huang, George Q. Li

**Affiliations:** ^1^College of Medical Technology, Chengdu University of Traditional Chinese Medicine, Chengdu, China; ^2^Faculty of Medicine and Health, The University of Sydney School of Pharmacy, The University of Sydney, Sydney, NSW, Australia; ^3^Faculty of Medicine, Department of Pharmacology and Therapy, Udayana University, Jimbaran, Indonesia; ^4^Faculty of Engineering and Information Technologies, Centre for Advanced Food Enginomics, The University of Sydney, Sydney, NSW, Australia; ^5^Centenary Institute of Cancer Medicine and Cell Biology, The University of Sydney, Sydney, NSW, Australia; ^6^NICM Health Research Institute, Western Sydney University, Penrith, NSW, Australia; ^7^School of Life Science, Beijing Institute of Technology, Beijing, China; ^8^School of Pharmaceutical Sciences, Sun Yat-sen University, Guangzhou, China; ^9^School of Pharmacy, Chengdu University of Traditional Chinese Medicine, Chengdu, China; ^10^The National Center for Natural Products Research, The University of Mississippi, Oxford, MS, United States; ^11^Institute of TCM-related Comorbidity, Nanjing University of Chinese Medicine, Nanjing, China; ^12^Chengdu Tepu Biotech Co., Ltd., Chengdu, China; ^13^College of Fundamental Medicine, Chengdu University of Traditional Chinese Medicine, Chengdu, China; ^14^Innovative Institute of Chinese Medicine and Pharmacy, Chengdu University of Traditional Chinese Medicine, Chengdu, China

**Keywords:** mechanism, active compounds, dendrobiums, polysaccharides, bibenzyls, diabetes, gut microbiota

## Abstract

**Background:**

Medicinal dendrobiums are used popularly in traditional Chinese medicine for the treatment of diabetes, while their active compounds and mechanism remain unclear. This review aimed to evaluate the mechanism and active compounds of medicinal dendrobiums in diabetes management through a systematic approach.

**Methods:**

A systematic approach was conducted to search for the mechanism and active phytochemicals in Dendrobium responsible for anti-diabetic actions using databases PubMed, Embase, and SciFinder.

**Results:**

Current literature indicates polysaccharides, bibenzyls, phenanthrene, and alkaloids are commonly isolated in *Dendrobium* genusin which polysaccharides and bibenzyls are most aboundant. Many animal studies have shown that polysaccharides from the species of *Dendrobium* provide with antidiabetic effects by lowering glucose level and reversing chronic inflammation of T2DM taken orally at 200 mg/kg. *Dendrobium* polysaccharides protect pancreatic β-cell dysfunction and insulin resistance in liver. *Dendrobium* polysaccharides up-regulate the abundance of short-chain fatty acid to stimulate GLP-1 secretion through gut microbiota. Bibenzyls also have great potency to inhibit the progression of the chronic inflammation in cellular studies.

**Conclusion:**

Polysaccharides and bibenzyls are the major active compounds in medicinal dendrobiums for diabetic management through the mechanisms of lowering glucose level and reversing chronic inflammation of T2DM by modulating pancreatic β-cell dysfunction and insulin resistance in liver as a result from gut microbita regulation.

## Introduction

### Diabetes and Diabetes Management

Diabetes is a chronic disease resulted from insufficient insulin produced by the pancreas or due to ineffective function of produced insulin. The uncontrolled blood sugar over time leads to serious damage to the body systems and contributes to the severity and mortality of diabetic patients. Type 2 Diabetes Mellites (T2DM) is one of the major chronic non-communicable diseases and a metabolic disorder that occurs owing to insulin resistance and the subsequent failure to secrete sufficient insulin by the pancreas. This progressive disease is characterized by the high level of blood glucose and mostly leads to microvascular and macrovascular complications (1).

Chronic hyperglycemia induces various cellular metabolic mechanisms leading to the accumulation of reactive oxygen species (ROS) and advanced glycation end products (AGEs). This condition triggers another cascade metabolic response that secretes a wide range of pro-inflammatory cytokines such as Tumor Necrosis Factor (TNF) α, interleukin (IL)-6, IL-8 and activates nuclear factor κB (NF-κB) cascades, resulting in the release of more cytokines, expression of adhesive molecules, and leucocyte activation (2, 3). Additionally, ROS upregulates matrix metalloproteinases (MMPs), which belong to the protein family of multifactor responsible for atherosclerosis (4).

Currently oral anti-diabetic drugs are available to control blood glucose levels but unable to halt the progression of chronic inflammation. The side effects such as digestive disturbance, severe hypoglycemia, myocardial infarctions and risk of fractures in women were reported to be associated with oral anti-diabetes treatments (5–8). Hence, there is an urgency to develop a new approach to modulate the disease and its vascular complications.

### Herbal Medicines and TCM

Traditional Chinese medicine (TCM) is a system of traditional medicine that is based on the theories, and practices of Chinese culture. It includes many practices such as acupuncture, Chinese therapeutic massage, and herbal medicine, and is characterized by a holistic understanding on the interaction between humanity and nature and the impact of internal and external energy flow on human body (9). Significant high-quality research has been conducted on herbal medicine for new drug discovery and development and to understand the mechanism of action for the treatment of major public health problems and chronic non-communicable diseases such as cancer, metabolic syndrome, and diabetes mellitus (9–11). In TCM, diabetes is referred to *Yin* deficiency and dryness-heat can be treated with Chinese herbs and formulas of corresponding functions (11). Phytochemicals such as polysaccharides, saponins, alkaloids, flavonoids, and terpenes of herbal medicines are shown to be active compounds in diabetes treatment through several mechanisms of action, such as lowering blood glucose level, increasing insulin sensitivity, and inhibiting α-glucosidase activity (12). However, for most herbs, active compounds and mechanisms of action are not well-defined.

### Medicinal Dendrobium and Clinical Studies

Dendrobiums is a common name for dendrobium orchids and medicinal dendrobiums. Dendrobiums have been used as traditional medicine for thousand years in China. They have been traditionally used to enhance immunity, lowering blood glucose level, and as a gastric tonic. While in Australia, they have been used for dysentery, relieve pain, allergy rash, and itchy skin (13, 14).

**Figure 1:**
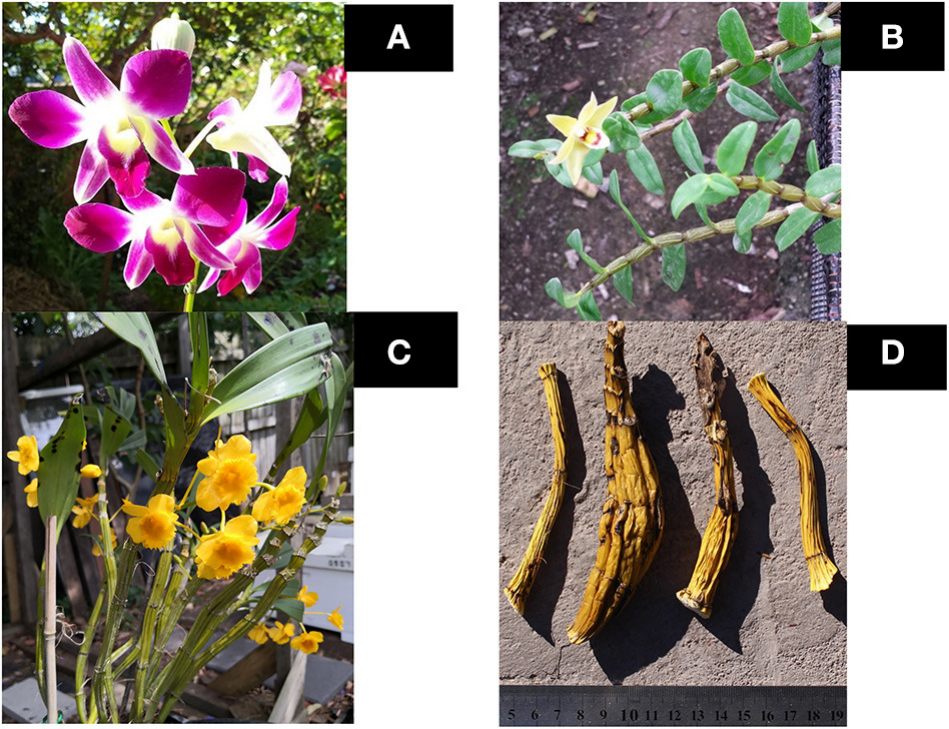
Representative species of *Dendrobium* genus. **(A)** *Dendrobium nobile*; **(B)** *D. officinale*; **(C)** *D. chrysotoxum*; **(D)** Dry herb of *D. chrysotoxum*.

*Dendrobium* containing over 1,000 species, is one of the largest genera in the Orchidaceae family. The Chinese Pharmacopeia lists two monographs of dendrobium herb, Dendrobiii Caulis (Shi Hu), including *D. nobile* Lindl.; *D. chrysotoxum* Lindl., *D. fimbriatum* Hook. and close species; and Dendrobii Officinalis Caulis (Tiepishihu), for *D. officinale* Kimura et Migo (15) (Figure 1). *D. nobile* and *D. officinale* belongs to *Dendrobium* sect. *Dendrobium. D. chrysotoxum* belongs to the *Dendrobium* sect. *Densiflora* Finet. while *D. fimbriatum* belongs to the *Dendrobium* sect. *Holochrysa* Lindley (16). The monographs in the Chinese Pharmacopeia are defined by botanical and pharmacognostic characters and chemical determinations which include polysaccharides for *D. officinale*, dendrobine alkaloids for *D. nobile*, erianin for *D. chrysotoxum*, and phenolic patterns on thin-layer chromatography (TLC) for both *D. chrystoxum* and *D. fimbriatum*. Although the chemical components are used as marker compounds, they are not clearly defined to differentiate species, quality standards and biological activities.

The monographs have similar TCM properties and channel tropinism as sweet, slightly cold, entering stomach and kidney channels. Their functions are beneficial to stomach and essence, tonifying *Yin* and clearing heat (15). Based on traditional usage in the previous studies, *Dendrobium* spp. has been applied to treae lung, stomach, and kidney deficiency syndrome. This syndrome is differentiated with the symptoms of increasing thirsty, frequent urination, gums bleeding which are showing in *Xiao Ke* patients (14). In the TCM, the clinical function of the dendrobium is suggested as a promoter of producing body fluids, nourishing *Yin*, and then showing antipyretic efficacy (15, 17, 18). Interestingly, in Australia *Dendrobium species* has a written record of their application of treating allergic skin rash and itchiness, and using as bush food (19). Randomized clinical trials suggest *Dendrobium* has anticancer activity and prevent liver damage via its antioxidant property. The herb can tonify *Yin* by enhancing immunity, promoting digestive function, and increasing salivary secretion (20). Clinical studies have reported that the application of *Dendrobium species* provides a considerable potential to treat diabetes of human beings via reducing the risk of developing diabetes and its fatal complications. It inhibits the develop the ketoacidosis in type I diabetes patients (21) and suppresses the development of retinopathy (22) and reduces the risk of myocardiopathy in diabetic patients (23). Treatment with Dendrobium could inhibit the development of non-small cell lung cancer and nasopharyngeal carcinoma (24, 25). Other studies suggest the application of *D. huoshanense* beneficial to treat atopic dermatitis papatientsia suppressing serum expression of IL-5, IL-13, Tumor Growth Factor (TGF) β 1, and Interferon (IFN) γ from the victims of atopic dermatitis (26). Limited studies indicate the herb is relatively safe. Acute and subchronic toxicity on *Dendrobium* aqueous extract in Sprague-Dawley rats at 2,400, and 5,000 mg/kg did not result in any toxic effects (27, 28).

### Literature Search and Aim of the Article

Previous reviews on *Dendrobium* species have provided a comprehensive overview on their chemical components and bioactivities including anti-cancer, anti-diabetic, anti-inflammatory, and neuroprotective effects, without a particular focus on the mechanism of action and active compounds related to diabetes (29–31). Phytochemicals such as polysaccharides, bibenzyls, and alkaloids have been used in the quality standards of dendrobium monographs although they are used as marker compounds, rather than active compounds. Focused review on the mechanism and active compounds of dendrobiums related to diabetes is still lacking. Therefore, this review aimed to evaluate the mechanism and active compounds of *Dendrobium* in diabetes management through a systematic approach. The keywords used in the literature search were “*Dendrobium*,” “polysaccharides,” “bibenzyl,” “phenanthrenes,” and “diabetes,” using PubMed, Embase, and SciFinder which have covered the publications up to 2021.

## Chemistry of *Dendrobium*

**Figure 2:**
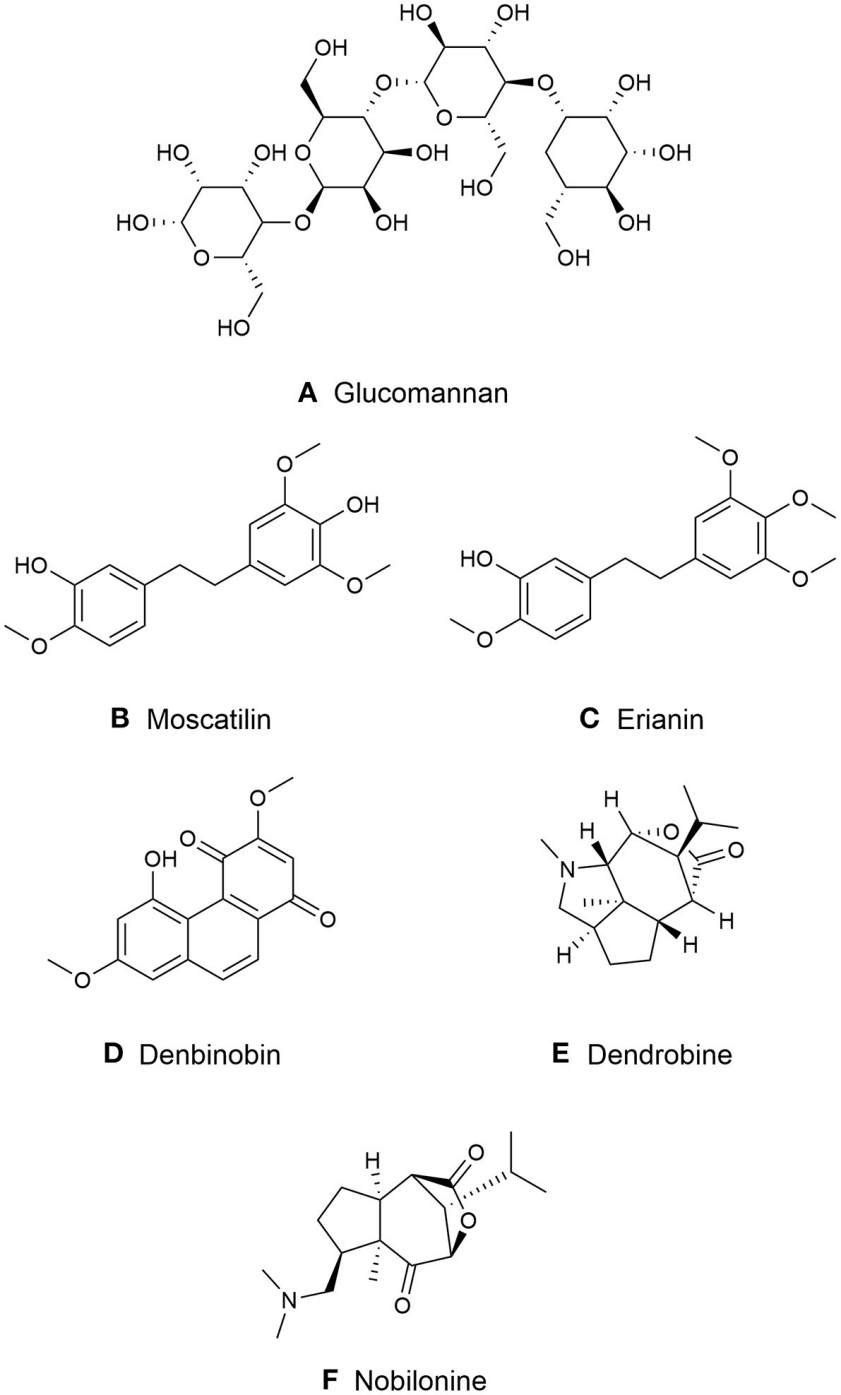
Major chemical compounds in medicinal *Dendrobiums*. **(A)** Glucomannan; **(B)** Moscatilin; **(C)** Erianin; **(D)** Dendrobine; **(E)** Denbinobin; **(F)** Nobilonine.

*Dendrobium* species vary in chemical profiles. Various species may contain polysaccharides, alkaloids and substantial aromatic compounds including bibenzyls, fluorenones, phenanthrenes and sesquiterpenoids, coumarins (29). The representative compounds are shown in Figure 2.

### Polysaccharides

Polysaccharides are macromolecules of carbohydrate consisting of long chains of numerous monosaccharides linked by glycosidic linkages. The constituents and linkage with monosaccharide in the medicinal plant determine the bioactivity of the polysaccharides in immunomodulatory, anti-inflammatory, antioxidant, antitumor, and antidiabetic effects (32). *Dendrobium* contains a significant amount of polysaccharide composed of the monosaccharides such as glucose, galactose, mannose, xylose, arabinose, rhamnose, glucuronic acid, and galacturonic acid (33).

Two polysaccharides are reported in *D. officinale* namely as *Dendrobium* polysaccharide (DOP)a and DOPb with a M_w_ of 8.1 × 10^5^ and 6.7 × 10^5^Da, which are mainly composed of mannose and glucose. Both DOPa and DOPb have a backbone of 1,4-β-D-mannopyranosyl and β-D-glucopyranosyl residues (34). The glucomannan with a M_w_ of 8,500 Da is extracted from *D. officinale* that is composed of mannose, glucose, arabinose, and galacturonic acid at molar ratio of 6.2:2.3:2.1:0.1 linked by the backbone of (1 → 4)-β-D-mannopyranosyl and (1 → 4)-β-D-glucopyranosyl residues (35). The O-acetylated glucomannan is a purified polysaccharide DOP-1-1 extracted from *D. officinale*. It is composed of mannose and glucose at a ratio of 5.9:1 with M_w_ of 1.78 × 10^5^ Da (36). The 2-O-acetylglucomannan with (1 → 4)-β-D-Manp and β-D-Glcp backbone is composed of mannose, glucose and arabinose at ratio of 40.2:8.4:1 with M_w_ of 1.3 × 10^5^ Da (37).

The polysaccharides found in *D. nobile* are *D. nobile* polysaccharide namely as DNP that are consisted of rhamnose, arabinose, xylose, mannose, glucose, and galactose linked by (1 → 4)-α-D-Glcp and (1 → 6)-α-D-Glcp at a ratio of 1.00:2.80:2.20:30.76:117.96:31.76 (38). Four derivatives DNP1-1, DNP2-1, DNP3-1, and DNP4-2 from *D. nobile* are composed of the similar monosaccharides of mannose, glucose, galactose and rhamnose, arabinose, and xylose with the M_w_ of 1.36 × 10^5^ Da, 2.77 × 10^5^ Da, 1.18 × 10^5^ Da, and 1.14 × 10^5^ Da, respectively (39).

A water-soluble polysaccharide with M_w_ of 7.3 × 10^5^ is extracted from *D. huoshanense*. It is mainly composed of glucose, xylose, galactose, and galacturonic acid linked by the 1,6-β-D-Glcp, 1,4- β-D-Glcp, and 1,4,6-β-D-Glcp (Figure 2A) (40).

### Bibenzyls

Bibenzyl is known as 1,2-diphenylethane that is a class of phenolic compounds in many *Dendrobium* species. The phenolic compounds are popular and at a concentration up to 4 mg/g are often used as chemical markers in *Dendrobium* (41). Moscatilin (dendrophenol) and gigantol are two most common bibenzyls found among *Dendrobium* species and may be detected in almost all *Dendrobium* species (41, 42).

Moscatilin is formerly purified from *D. moscatum*, an India orchid (43). Its chemical structure is identified as 4,4′-dihydroxy-3,3′,5-trimethoxybibenzyl with molecular formula C_17_H_20_O_5_ that is structurally similar to erianin (43) (Figure 2B). Yang et al. reported that the highest concentration of moscatilin was found in *Dendrobium brymerianum* Rchb.f. (41).

Erianin initially purified from orchid *Eria carinata* Gibson ex Lindl. is limited and mainly presented from *D. chrysotoxum*, (43, 44). The molecular formula of erianin is C_18_H_22_O_5_ with a structure 3-hydroxy-3′,4′,4,5′-tetramethoxylbibenzyl (Figure 2C). It is closely related to combretastatins (CA-4), a polyphenolic *cis*-stilbene extracted from *Combretum caffrum* (Eckl. & Zeyh.) Kuntze (44) that is differentiated from a double bond between erianin and CA-4.

The substituents of bibenzyls often take place at the para and/or meta-positions on the benzene ring while the C7 and C8 atoms are rarely substituted. Common substituents include hydroxyl and methoxy but mono-substitution of bibenzyls has not been identified (29). Three main bibenzyl compounds erianin, moscatilin and gigantol are similar in structure but can be differentiated by the positions of methoxy and hydroxyl groups on the phenyl rings. Erianin has been reported with highly potent anticancer activity against cancer cell line.

### Phenanthrenes

Phenanthrenes is another phenolic compound, also known as stilbene and diterpenoid-derived compounds. They have been mainly identified from higher plants, including *Dendrobium*. Phenanthrenes are categorized into three groups: monophenanthrenes, diphenanthrenes and triphenanthrenes (45). Various phenanthrenes derivatives have been isolated from *Dendrobium* species. Most of them occur in monomeric form with hydroxy and or methoxy substituted with equally 9,10-dihydro phenanthrenes derivatives. Phenanthraquinones (another type of monomeric phenanthrenes), such as denbinobine and moniliformine found in *D. nobile* and *D. moniliforme* (46, 47). Denbinobine was reported to demonstrate anti-cancer activity on leukemic, prostate cancer and gastric cancer cell (47–49) (Figure 2D).

### Alkaloids

In contrast to bibenzyls, dendrobine was the first alkaloid identified from *D. nobile* (50). So far, fourteen alkaloids have been isolated from *D. nobile*, which are the largest number of alkaloids among *Dendrobium* species (29).

The molecular formula of dendrobine is C_16_H_25_O_2_N, which contains a N-methyl group with one picrotoxan-type sesquiterpenoid combined with a five-membered C2-C9-linked N-heterocycle and a C3-C5-linked lactone ring as the basic skeleton (50, 51) (Figure 2E). Other major alkaloids, nobilonine-type sesquiterpenoid (C_17_H_27_O_3_N), possess an opened C2-C9 N-heterocycle ring between C2 and N and form a ketone at C-2 (51, 52) (Figure 2F). Moniline (C_18_H_29_NO_4_) is another alkaloid isolated from *D. moniliforme* which contains α, β-unsaturated ketone at correlations of H-9/C-10 and H-9/C-8 (53).

## Mechanisms and Active Compounds of *Dendrobium* For Diabetes

### *Dendrobium* Polysaccharides for Glycemic Control and Diabetic Complications

Polysaccharides of *Dendrobium* are more effective in reducing glucose level. Comparison study of four *Dendrobium* species on Kunming mice showed that polysaccharides from *D. huoshanense, D. chrysotoxum, D. officinale*, and *D. nobile* reduced blood glucose level at dosages of 50, 100, and 200 mg/kg. This effect is comparable to 200 mg/kg of metformin. Moreover, all species except *D. chrysotoxum* reduced glycosilated serum protein level and increased serum insulin level (54). Other studies also reported similar effects of *D. huoshanense, D. chrysotoxum*, and *D. officinale* polysaccharides on blood glucose level (55–57). One study reported that polysaccharides of *D. officinale* at dose 1 g/kg had no effect on the blood glucose levels of STZ-induced diabetic mice. Interestingly, this experiment simultaneously suggested that the treatment considerably reduced the levels of total cholesterol, tryglyceride, BUN, creatinin and prevented hypoalgesia, indicating its potential in preventing complications of T2DM (58) (Table 1).

**Table 1:** Glycemic control activity of *Dendrobium*.

**Active compound**	***Dendrobium* species**	**Model**	**Dosage**	**Parameters**	**Reference**
Polysaccharide	*D. huoshanense*	STZ induced of Sprague-Dawley rats	50, 100 and 200 mg/kg, orally	Blood glucose level ↓ in dose-dependent manner. The lowest level was with 200 mg/kg	(55)
	*D. huoshanense*	Alloxan induced of Kunming mice	50, 100, and 200 mg/kg, orally	Blood glucose level ↓ Glycosilated serum protein level ↓ Serum insulin level ↑ Number of islet cell ↑	(54)
	*D. chrysotoxum*	Alloxan induced of BALB/c mice	200 and 500 mg/kg	Blood glucose level ↓	(57)
	*D. chrysotoxum*	Alloxan induced of Kunming mice	50, 100, and 200 mg/kg, orally	blood glucose level ↓ on middle and high dose No effect on glycosilated serum protein & serum insulin level Number of islet cell ↑	(54)
	*D. nobile*	Alloxan induced of Kunming mice	50, 100, and 200 mg/kg, orally	Blood glucose level ↓ Glycosilated serum protein level ↓ Serum insulin level ↑ Number of islet cell ↑	(54)
	*D. officinale*	Alloxan induced of Kunming mice	50, 100 and 200 mg/kg, orally	Blood glucose level ↓ Glycosilated serum protein level ↓ Serum insulin level ↑ Number of islet cell ↑	(54)
	*D. officinale*	STZ induced of Kunming mice	75, 150 & 300 mg/kg	Fasting blood glucose level ↓ on medium and High dose Level of triglyceride and total cholesterol ↓	(56)
Phenanthrene Loddigesiinols G-J	*D. loddigesii*	α-Glucosidase assay	10 μmol/mL	Inhibit α-Glucosidase activity	(59)

Hyperglycemia and insulin resistance are believed to activate inflammation cascades that lead to microvascular and macrovascular complications. Increased AGE-product along with the secretion of pro-inflammatory cytokines and activated NF-κB pathway are implicated in the pathogenesis of diabetic complications (60). The sulfated polysaccharide from *D. huoshanense* at dosage 1.0 mg/mL gave an inhibition up to 72.54% in the formation of amadori products during 3 weeks culture time while that of aminoguanidine at the same dosage was halved in the formation of amadori products.

The AGEs formation is inhibited by 51.9% during 28-day treatment and is slightly higher than the inhibition by aminoguanidine (54). A water-soluble polysaccharide from *D. huoshanense* gives inhibitory activity on protein glycation by 23% at the concentration of 0.5 mg/mL. This effect is comparable to 0.3 mg/mL vitamin C, which inhibits protein glycation by 28% (40). Furthermore, the polysaccharides of *D. huoshanense* is also reported on inhibition of AGEs formation in the lenses of streptozotocin-induced diabetes cataract mice by 34.9–40.2% at three different dosages of 50, 100, and 200 mg/kg. It is also reported that the reduction of NO and NOs levels is 50% lower than that of the mice being treated with STZ only, especially at 200 mg/kg. Blood sugar levels in the mice treated with polysaccharides are decreased significantly on a dose-dependent manner (55).

### *Dendrobium* Polysaccharides Alleviates Dysfunction of Pancreatic Cells

As a chronic metabolic disease, T2DM has a complex pathogenic mechanism in which chronic hyperglycemia is its signature feature. Pancreatic β-cell dysfunction and insulin resistance in the target organs are the core mechanisms of metabolic disorders in diabetes mellitus. β-cell dysfunction may be caused by both lipotoxicity and glucotoxicity. If the free fatty acids (FFA) enter to the pancreatic β-cells and can't be decomposed in time the lipotoxicity will result in accumulation of excessive lipids that then leads to apoptosis of β-cells. In addition, the chronic hyperglycemic environment resulting from insulin resistance and insulin hypersecretion may damage pancreatic β-cells and further aggravate the insulin secretion defect.

DOP is reported to reduce glucagon level that interferes signaling pathway of hepatic glycogen metabolism, including the cAMP-PKA and Akt-FoxO1 pathway (61). Modulation on several proteins, including HOMA-β, HOMA-IR, Glucagon-like peptide-1 (GLP-1), insulin receptor substrate 1 (IRS-1), phosphoinositide 3-kinase (PI3K), and Akt are reported on diabetes with suppression of apoptosis on pancreatic cells (62). Several studies have shown that DOP can exert a protective effect on β-cells (61). Oral administration of DOP may restore the morphology of pancreatic cells and increase the proportion of pancreatic β cells. The increase of GLP-1 concentration in diabetes mellitus model mice suggests that the protection by DOP may be related to the induced anti-apoptotic effect (62).

GLP-1 is secreted by intestinal L cells that is involved in promoting insulin secretion and inhibiting glucagon secretion. GLP-1 delays gastric emptying and increases satiety signals in the brain without causing hypoglycemia as a side effect (63). It is an important target for new drug development in type 2 diabetes in recent years (64). GLP-1 achieves the anti-apoptotic effect by upregulating the expression of the anti-apoptotic factor (BCL-2) or downregulating the expression of the pre-regulatory death factor (caspase-3) (65).

The oral administration of DOP stimulates GLP-1 secretion and effectively inhibits apoptosis of pancreatic β-cells in T2D mice, exerting a protective effect on pancreatic β-cells and improving cells function (62). The murine intestinal endocrine cell line STC-1 is a GLP-1 producing cell line, and DOP also induces GLP-1 production by STC-1 secretion, and inhibition of the Ca^2+^/CaM/CaMKII and MAPK pathways counteracts this facilitation, suggesting that this pathway may be involved in intracellular DOP-induced GLP-1 secretion (66). Considering the way polysaccharides are utilized in the intestine, we hypothesize that DOPs stimulate GLP-1 secretion by enteroendocrine cells.

### *Dendrobium* Polysaccharide Modulates Insulin Signaling and Alleviates Insulin Resistance

Promoting glycogenesis and inhibiting gluconeogenesis are the main functions by insulin in regulating blood glucose. Insulin regulates a variety of downstream effector such as liver, skeletal muscle, and white adipocytes, via binding to the insulin receptor (INSR) on the plasma membrane of target cells to exert all its known physiological effects (67). Activated INSR initiates downstream metabolic signaling by recruiting phospho-tyrosine-binding scaffold proteins. IRS1 and IRS2 belonging to the IRS family are considered to mediate most of the metabolic effects of INSR activation (67).

A homogeneous polysaccharide galactose (GXG) purified from *D. huoshanense* promotes IRS-1 tyrosine phosphorylation in the liver of T2DM mice, restoring INSR function and improving insulin resistance (62).

The glycogen synthesis pathway in hepatocytes is influenced by insulin regulation of glycogen synthase (GS) and glycogen phosphatase (GP) (68). Insulin-stimulated Akt activation may regulate GS function by inhibiting GSK3 activity and also regulate glycogen synthase degeneration by newly synthesized glucose-6-phosphate as a way to promote glycogen synthesis (69). Activation of AKT via receptor binding by insulin also phosphorylates the regulatory transcription factor Forkhead Box O1 (FoxO1) to disable its transcription factor activity, thereby inhibiting the transcription of gluconeogenesis-related genes. Insulin resistance is commonly accompanied with the enhanced gluconeogenesis and increased blood glucose levels mediated by the activation of FoxO1 (70).

It has been shown that DOP reduces hepatic glucose levels in T2DM mice by strengthening hepatic glycogen synthesis (61). Meanwhile, oral administration of GXG that reduced the sensitivity of the model mice to the pyruvate test due to its function by controlling the blood glucose may be related to the inhibition of gluconeogenesis (62). Oral administration of GXG significantly enhances PI3K and Akt phosphorylation and enhances insulin signaling in hepatocytes in the model mice. Moreover, GXG promotes islet-mediated glycogen synthesis by inhibiting GSK3β phosphorylation (62). *D. officinale* water extract (DOWE), with O-acetylglucomannan as the main component, also reduces blood glucose levels by promoting hepatic glycogen synthesis in the diabetic mice (71).

Insulin inhibits lipolysis via cAMP and protein kinase A (PKA) (72). In addition, the cAMP/PKA signaling pathway can regulate glucose homeostasis at multiple levels, including insulin and glucagon secretion, glucose uptake, glycogen synthesis and catabolism, gluconeogenesis, and neural control of glucose homeostasis (61).

DOP can remove lipid deposits from hepatocytes that may be related to its function in regulating lipid metabolism (73). At the same time, DOP significantly inhibits the glucagon-mediated cAMP-PKA signaling pathway, which further increases the expression of GS and decreases the expression of GP, thus promoting hepatic glycogen synthesis and inhibiting hepatic glycogen degradation in diabetic mice. DOP reduces the expression of AKT downstream factors G6Pase and PEPCK, which inhibits hepatic gluconeogenesis in diabetic mice through the pancreatic glucagon mediated Akt/FoxO1 signaling pathway (61). In addition, the same study demonstrats that DOP treatment improves the stability of hepatic glycogen alpha particles in model mice, making them more tightly bound between beta particles and less susceptible to catabolism, which could alleviate the accelerated glycogen breakdown caused by diabetes.

### *Dendrobium* Polysaccharide Alleviates Lipid-Induced Insulin Resistance

The chronic environment of diabetes-mediated nutrient overload aggravates the accumulation of lipids in the liver. Lipids are stored primarily as relatively inert triglycerides, and triglycerides stored in WAT are unlikely to directly affect insulin function by themselves (74), but increase levels of the bioactive lipid diacylglycerol (DAG). The protein kinase C-ε (PKCε) activated by DAG can directly inhibit INSR function, which in turn triggers insulin resistance (75). A large number of lipid droplets are visible in the liver tissue of diabetic rats, and administration of DOP can effectively reverse hepatic steatosis (62). Omics analysis of the diabetic rat liver tissues from diabetic rats indicates that T2DM significantly altered the metabolic pattern of the rat liver and that DOP intervention alleviated this metabolic disorder (76).

DOP treatment alleviates the extent of disturbances in the metabolism of fatty acids, glycerolipids (diacylglycerol and triacylglycerol), and glycerophospholipids (phosphatidylcholine and phosphatidylethanolamine) in diabetic rats. The levels of fatty acids (FAs) and glycerophospholipids are typically lower in the DOP-treated group compared to the diabetic model rats. SFAs can act as substrates for ceramide biosynthesis (77), and ceramides, in turn, reduce AKT activity by acting on Protein Phosphatase 2A (PP2A) or PKCζ or through activation of inflammatory mediators (e.g., TNF-α) thus leading to insulin resistance (78). Furthermore, the SFA-TLR4 signaling pathway also mediates the development of insulin resistance by upregulating the transcription of ceramide biosynthetic enzymes (79). DOP treatment significantly alleviates the accumulation of saturated fatty acids (SFA's) and unsaturated fatty acids (UFAS) in the liver. UFAs are deemed as activators of the innate immune component Toll-like receptor 4 (TLR4) and this modulatory effect may be associated with the function of Dop in alleviating insulin resistance.

The COX-2 PGE_2_-EP3-mediated signaling in adipocytes induces the production of inflammatory factors and mediates insulin resistance (80). Levels of prostaglandins, which have pro-inflammatory effects, are significantly elevated in diabetic rats, while the supplementation of DOP significantly reduces their concentrations in the liver. In addition, many phospholipids are involved in the hepatic DAG synthesis process, possibly through the DAG/PKC pathway-mediated insulin resistance, which has been suggested to be a key component associated with IR and T2DM (81). Phosphatidylethanolamine (PE) and phosphatidylcholine (PC) concentrations are significantly increased with DOP treatment, which may be related to the improvement of the synthesis of VLDL by DOP (73).

In conclusion, DOP modulates disturbances in lipid metabolism to alleviate lipid-induced insulin resistance although its targeting effect is not clear. Disturbances in lipid metabolism in the diabetic environment are a very complex pathological process. There is evidence that triglycerides alone are unlikely to directly affect insulin function (82), and we believe that regulation of DAG levels to alleviate insulin resistance may be one of the important channels for DOP to restore hepatic lipid metabolism disorders. On the other hand, a metabolic analysis reveals that DOP treatment balanced the metabolism of ceramide and bile acids, including deoxycholic acid, taurocholic acid, and bile acids (76).

Bile acids (BAs) are steroid molecules synthesized in the liver from cholesterol, and their signaling pathways significantly affect the metabolic regulation of T2DM. An increase in hydrophobic bile acids may impair glucose homeostasis by promoting inflammation and endoplasmic reticulum stress (83). The endoplasmic reticulum (ER) manifests as the unfolded protein response (UPR). The key effector of the UPR includes c-Jun NH2-terminal kinase (JNK) and the mRNA splice variant of X-box binding protein 1 (XBP1s). JNK potentially impairs proximal insulin signaling directly, whereas transcription of XBP1s could promote hepatic steatosis, which may further drive hepatic insulin resistance via DAG/PKCε signaling (78).

Bile acids are endogenous regulators of farnesoid X receptor (FXR) and disturbances in hepatic glucose metabolism facilitate bile acid synthesis, thereby affecting FXR-regulated β-cell glucose-stimulated insulin secretion and interfering with hepatopancreatic bile acid signaling (83). In addition, 12α-hydroxy bile acids, such as bile acids and deoxycholic acid, are negative regulators of insulin action in T2DM and may affect insulin signaling through the FoxO1/Cyp8b1 pathway (84, 85). Oral administration of DOP reduces bile acids in the liver of diabetic rats (76), implicating that DOP may be involved in the regulation of BA signaling and alleviate metabolic disorders in the diabetic setting, but its specific mechanism of action needs to be confirmed by further studies.

### *Dendrobium* Polysaccharides Regulate Oxidative Stress and Chronic Inflammatory Response

Hyperglycemia and insulin resistance are believed to activate inflammation cascades that lead to microvascular and macrovascular complications. The pathogenesis of diabetes complications is related to chronic inflammation. Several studies on *Dendrobium* species reveal their anti-inflammatory properties via the downregulation of pro-inflammatory cytokines and other related factors, suggesting the potential of *Dendrobium* species in preventing diabetic complications (46, 57, 86, 87).

Polysaccharides of *D. officinale* downregulate expression of pro-inflammatory cytokines and their associated factors such as TNF-α, IL-1β, TGF-β, fibronectin, and NF-κB at dosages 150 and 300 mg/kg on streptozotocin (STZ)-induced mice. It also reduces the activities of myocardial enzymes, creatine kinase (CK), and lactate dehydrogenase (LDH) at similar dosages.

These outcomes reflect the cardioprotective potency of polysaccharides isolated from *Dendrobium* species against diabetic cardiomyopathy (56). While in a diabetes mouse model, DOP shows reducing pro-inflammatory cytokines and at the same time decreasing the concentration of fatty acid and glycerophospholipids (76).

### Gut Microbiota Mediates the Antidiabetic Effect of *Dendrobium* Polysaccharides

In recent years, there are reports that diet may change the structure of gut microbiota. *D. officinale* could increase the abundance of *Akkermansia* and *Parabacteroides* in the T2DM mice (88). Meanwhile, the mixture of *D. officinale* and American ginseng could up-regulate the abundance of short-chain fatty acid (SCFA)-producing genera and probiotic genera including *Lactobacillus, Sutterella, Alistipes, Anaerovorax, Bilophila, Coprococcus, Gordonibacter*, Oscillibacter, among others, and reduces the abundance of *Collinsella, Rothia, Howardella, Slackia, Intestinibacter*, which are considered to be associated with the development of diabetes (89).

Polysaccharides, as a kind of polar macromolecules, are not easy to be absorbed by the human body through an oral intake under normal conditions. For a long time, the mechanism involved in the metabolism of polysaccharides in functional foods is not clear. Recent studies indicate the ability of the gut microbiota to use these insoluble fibers for energy and the resulting metabolites as signaling molecules or metabolic substrates that regulate host metabolism form the basis of a symbiotic relationship between the host and the resident gut microbiota.

SCFAs are the main catabolic products of this process, and they signal through G protein-coupled free fatty acid receptors (FFAR)2 and FFAR3 or function by regulating nuclear histone deacetylase (HDAC) activity (90, 91). It has been shown that DOP containing 1,4-β-mannopyranoside did not release free monosaccharides during metabolism *in vivo* and *in vitro*. Only after the metabolism of DOP to SCFAs, it had an effect on host immune regulation, by inhibiting HDAC activity down-regulating the production of NF-κB and TNF (92).

Based on such phenomena, we suggest that the function of DOP in restoring metabolic disorders in diabetes is inextricably linked to the metabolism of the intestinal microbiota. However, DOP does not only alleviate diabetic symptoms through immunomodulatory effects mediated by SCFAs; restoration of islet cell dysfunction by stimulating GLP-1 secretion but is also one of the important mechanisms by which DOP exerts anti-diabetic activity. DOP exerts a protective effect on the pancreatic islet β-cells by promoting GLP-1 secretion (62). SCFA signaling in the small intestine is mainly stimulated by FFAR3, while in the colon FFAR2 can mediate GLP-1 release from colonic L cells (93). In addition to indigestible carbohydrates, the intestinal microbiota can metabolize a number of substrates present in the intestinal lumen.

Bacteria from the genera *Escherichia, Synechococcus*, and *Clostridium* can degrade tryptophan to indole, pyruvate, and ammonia using tryptase (94). Sulfate-reducing bacteria (SRB) can produce large volumes of hydrogen sulfide (H_2_S) in the colon (95), and both indole and hydrogen sulfide have shown to acutely stimulate GLP-1 secretion by GLUTag cells, a GLP-1-secreting cell line similar to L cells. Based on this evidence, further studies can be conducted to discern the specific mechanism by which DOP regulates GLP-1. Furthermore, there is evidence that oral DOP can modulate bile acid levels in the liver of rats in a diabetic model, suggesting that DOP treatment may promote bile acid synthesis and metabolism are closely linked to the function of microbiota. The intestinal lumen, BA, and the microbiota reciprocally regulate their composition (83).

**Figure 3:**
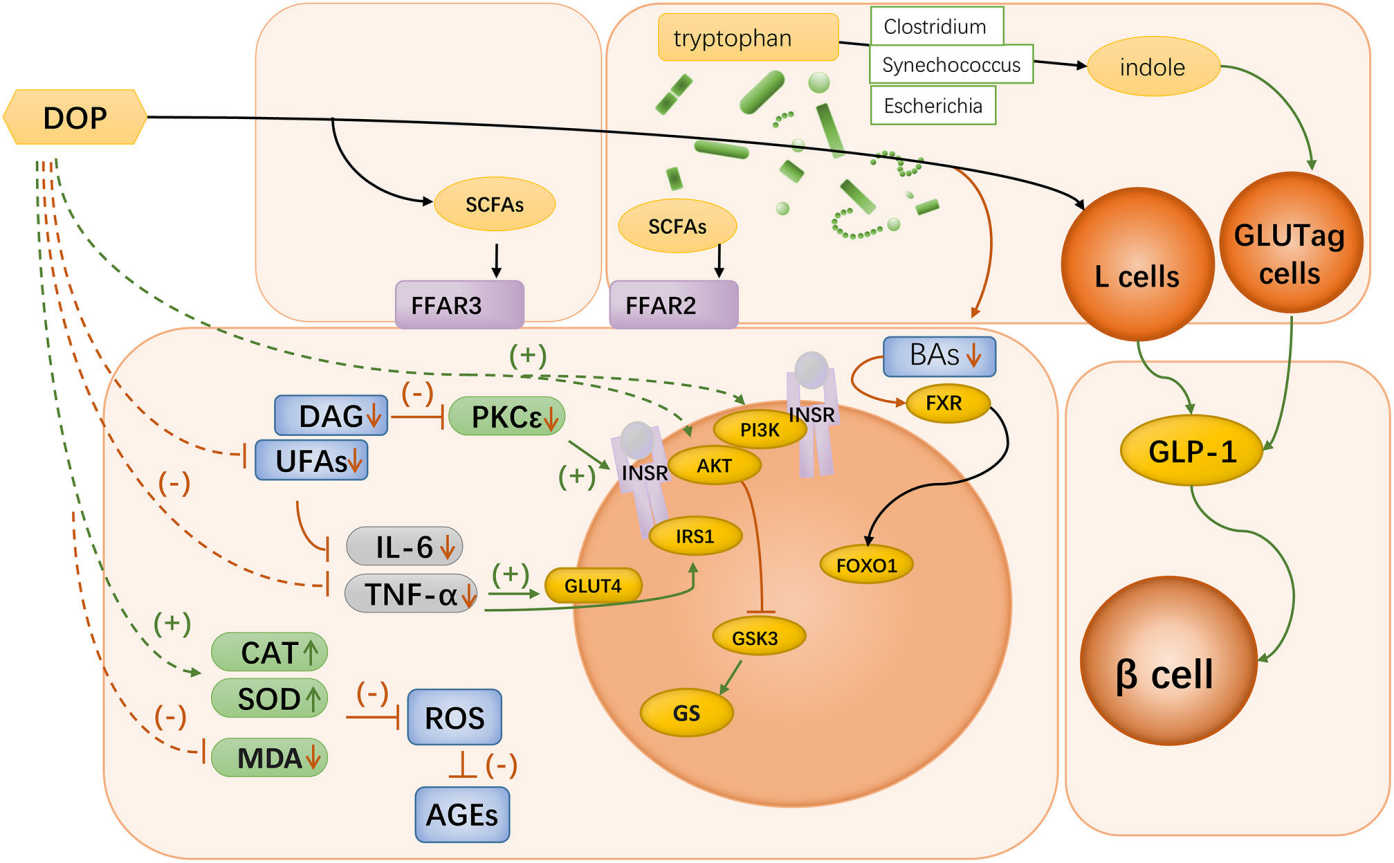
Mechanisms of *Dendrobium* polysaccharides in diabetes. Oral *Dendrobium* polysaccharides can be catabolized in the gut to produce SCFAs, which can bind to FFAR2 and FFAR3 in the small intestine and colon to participate in host immune regulation; Bacteria of the genera *Escherichia* spp., *Synechococcus* spp., and *Clostridium* spp. in the gut can degrade tryptophan to indole, which can acutely stimulate the function of GLUTag cells to secrete GLP-1. DOP can exert a protective effect on pancreatic β-cells by promoting GLP-1 secretion; DOP can be involved in BA signaling mediated by gut microbiota, which leads to improved insulin sensitivity through the regulation of FXR signaling; Oral administration of DOP can enhance the phosphorylation of PI3K and Akt in hepatocytes, which leads to the enhancement of insulin signaling intensity, and the regulation of GS function; Oral administration of DOP could improve the disorder of lipid metabolism in diabetic mice, and lowering the level of DAG could inhibit PKC activity and thus restore the function of INSR. UFAs could participate in immune regulation by activating TLR4, and DOP could alleviate the accumulation of UFAs in the liver; DOP treatment significantly reduced the concentrations of pro-inflammatory factors IL-6 and TNF-α and increased the level of IL-10 in the liver tissues of diabetic rats; DOP treatment up-regulated CAT and SOD levels and MDA levels in hepatocytes of diabetic rats, thereby scavenging excessive ROS and improving ROS-mediated accumulation of AGEs.

Bile acids are endogenous regulators of FXR. Metformin can inhibit intestinal FXR signaling in an AMPK-independent manner via the intestinal microbiota (96). The metformin specifically reduces the abundance of *Bacteroides fragilis* in the intestine, leading to an increase in FXR antagonists and resulting in increased insulin sensitivity (96). There is a possibility that DOP could play a similar role (Figure 3).

Overall, the current literature indicate that *Dendrobium* polysaccharides can restore the metabolic disorders caused by diabetes by repairing islet cell function, improving insulin resistance, and inhibiting oxidative stress and pro-inflammatory cytokines (Figure 3). However, the observed associations at this stage remain in observable appearance, and more evidence is needed to clarify a complete set of mechanisms of their actions. *Dendrobium* polysaccharide, as a macromolecule that cannot be directly utilized by the body, is not directly involved in the regulation of metabolism, and the gut microbiota might play a key role in explaining this linkage. DOP might be able to activate the secretion of GLP-1 by colonic L cells after metabolism by the gut microbiota in the colon. Further exploration of the mechanisms by which DOP mediates the involvement of the gut microbiota in short-chain fatty acid metabolism and further exploration of the mechanism of bile acid signaling by DOP could provide more evidence for the mechanism of action of *Dendrobium*.

### Anti-diabetic Complication and Anti-inflammatory Effects of Bibenzyls and Alkaloids

Erianin, a bibenzyl of *D. chrysotoxum*, downregulates vascular endothelial growth factor (VEGF) mRNA in *vitro* and *in vivo*. It also inhibits the PI3K phosphorylation pathway with its downstream molecules, AKT, mTOR, and P70S6 kinase. However, it shows no effect on blood glucose levels (97). Ethanol extract of *D. chrysotoxum* demonstrates anti-inflammatory properties on diabetic retinopathy SD mouse induced by STZ at three different doses of 30, 100, and 300 mg/kg. The mRNA expressions of VEGF and VEGF receptor 2 (VEGFR2) in the retina of diabetic rats are decreased in a dose-dependent manner in *D. chrysotoxum* treated groups. The expressions and serum levels of other inflammatory markers such as intercellular adhesion molecule 1 (ICAM-1), IL-6, and IL-1β are also reduced. Furthermore, two dosages of *D. chrysotoxum* (100 and 300 mg/kg) lower the serum level and mRNA expression of MMP2, with the highest dose decreasing the serum level and the mRNA expression of MMP9 (87).

A study conducted by Yu et al. revealed similar results in terms of anti-inflammatory properties of the ethanol extract of *D. chrysotoxum*. This study reported that the extract of *D. chrysotoxum* at similar dosages decreased the serum levels of TNF-α, IFN-γ, IL-8, IL-12, IL-2, IL-3, and IL-10 in diabetic rats (98). However, both studies reported that ethanol extract of *D. chrysotoxum* gave no effect on blood glucose level (87, 98). Further exploration in a recent diabetic mice revealed that erianin rescued the expression of occludin, claudin, and Iba-1 which had an essential role in diabetic retinopathy. The erianin is effetive on inhibition of signaling pathways, including ERK1/2 and NF-κB signaling (99) (Supplementary Table 1).

Diabetic patients have an increased risk of developing Alzheimer's disease. Chronic hyperglycemia increases amyloid-beta peptide production, which has a potential role in the pathogenesis and neurotoxicity of Alzheimer's disease (100). Alkaloid extract from *D. nobile* at doses of 80 and 160 mg/kg that contained dendrobine (90.7%), nobilonine (4.47%), dendramine (2.31%), and 3-hydroxy-2-oxodendrobine (1.29%) reduces expressions of NF-κB, TNFR1, and p38 MAPK in Alzheimer model rats induced by lipopolysaccharides (LPS). Expression of TNFR1 in the hippocampus is decreased significantly and this effect is comparable to ibuprofen (LPS: 1540; Alkaloid extract + LPS: 119; ibuprofen + LPS: 147). The alkaloid extracts treated rats also exhibited much better spatial memory than LPS treated rats, with searching distance and escape latency being 252 vs. 775 cm and 11.3 vs. 30.2 s, respectively (101).

Furthermore, the isolated alkaloid which contains 30.5% of dendrobine attenuates the phosphorylation of tau protein (at the sites of Ser199-202, Ser396, Ser404, Thr205, Thr231) and expression of GSK-3β on Alzheimer model rats induced with LPS. Apoptotic cells in the hippocampus due to LPS also decreases in the alkaloid treated group at doses of 20 and 40 mg/kg (102). Phenanthrene compounds have a great potent anti-inflammatory effect in cellular assays (Supplementary Table 2).

## Discussion and Conclusion

The literatures reviewed conclude that the mechanism by *Dendrobium* in diabetic management is complicated as the multiple active compounds are involved in directly and the gut microbita indirectly. This is the typical characters in many Chinese herbs. Therefore, it is greatly deserved to research and understand the mechanism by the herbs because they have been utilized in the assistance of human health for thousand years. This includes to know the distribution pattern and contents of the active compounds to build the platform for quality control of the herbs as well as which and how these compounds act in the specific clinical application.

Medicinal dendrobium is a unique Chinese medicinal herb including many species in one monograph with the same property and clinical application. Research on its underlying mechanism of actions and active compounds are critical to understand the traditional theory and provide scientific evidence for its clinical usage in diabetic management.

Current literature clearly indicates that phytochemicals have different distribution in species of *Dendrobium*. Polysaccharides, bibenzyls, phenanthrene and alkaloids are commonly isolated in the genus. However, their distributions and contents are variable. Polysaccharides are the major components distributed in the stems of all *Dendrobium*, although more concentrated in *D. officinale* and *D. nobile*. Bibenzyls and phenanthrenes are phenolic compounds which also present in many *Dendrobium* species in which the specific compounds such as erianin is mainly extracted from *D. chrysotoxum* (41, 42). Very few *Dendrobium* species such as *D. nobile* contain the compounds of alkaloids.

The major goals in T2DM treatment are to control blood glucose levels and prevent chronic micro and macrovascular complications. A number of researches conducted on animals have shown that polysaccharides from the species of *Dendrobium* at 200 mg/kg exert antidiabetic effects on lowering glucose level and reversing chronic inflammation of T2DM by modulating cytokines and other proteins that are responsible in inflammation cascade. It has been reported in many studies that *Dendrobium* polysaccharides protect pancreatic β-cell dysfunction and insulin resistance in liver as the key mechanisms of action. Recent studies reveal *Dendrobium* polysaccharides up-regulate the abundance of SCFA to stimulate GLP-1 secretion through gut microbiota. Further studies integrating animal modles and network pharmacology are warranted to confirm these mechanisms.

**Figure 4:**
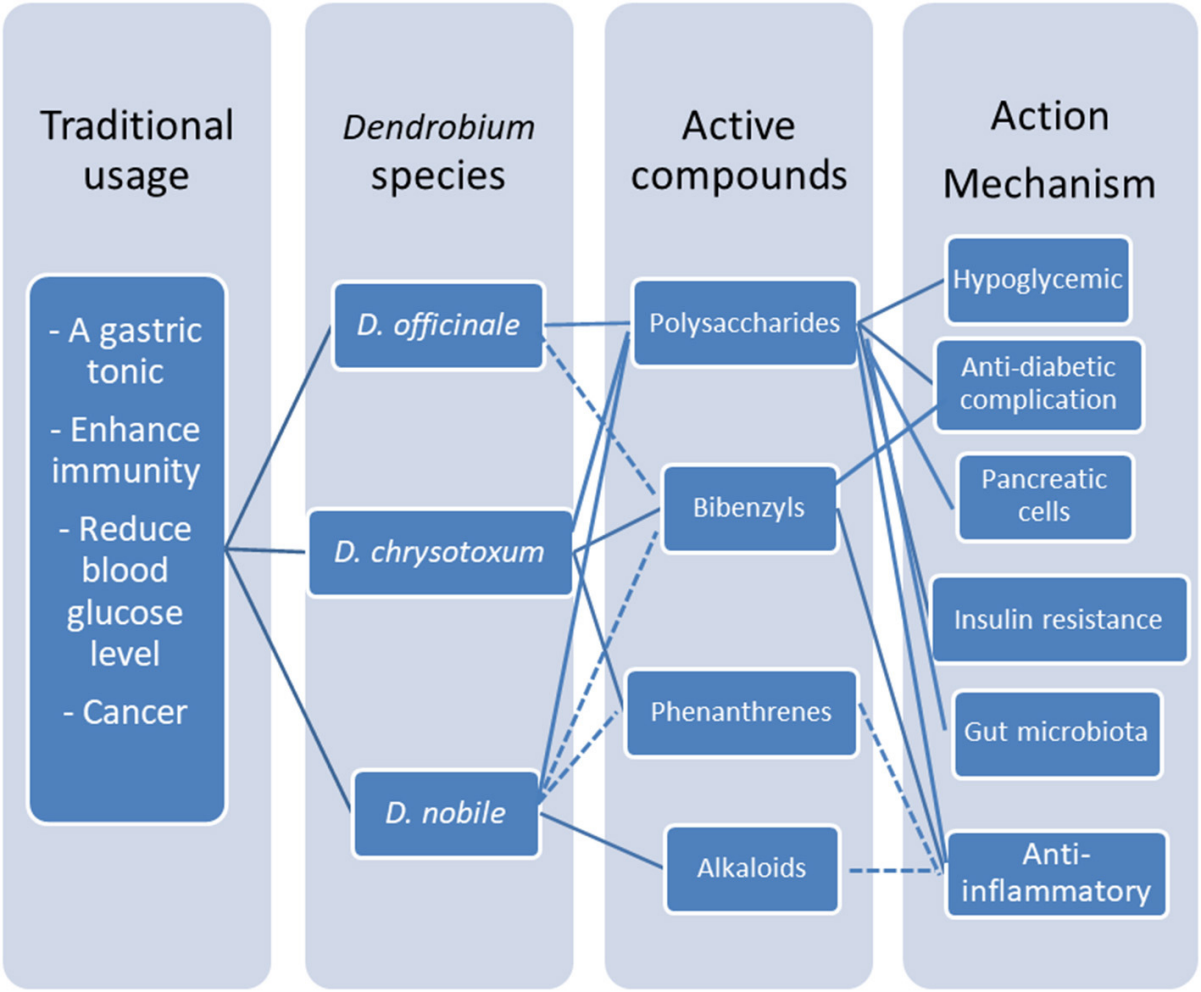
Multiple active compounds and multitargets platform of medicinal *Dendrobium*. Polysaccharides and bibenzyls are the major active compounds in medicinal dendrobiums for diabetic management, through multiple mechanisms including lowering glucose level and reversing chronic inflammation of T2DM by modulating pancreatic β-cell dysfunction and insulin resistance in liver, and indirectly via gut microbita.

Interestingly, erianin and gigantol, and phenanthrene do not have significant effect on the blood glucose level even at a dosage up to 100 μM but they possess the great potency to inhibit the progression of chronic inflammation in cellular studies. A limited pubilications report that *Dendrobium* alkaloid extracts have effect on inhibiting the progression of chronic inflammation in the Alzheimer model in rats (Figure 4).

In conclusion, polysaccharides and bibenzyls are the major active compounds in medicinal dendrobiums for diabetic management through the mechanisms of lowering glucose level and reversing chronic inflammation of T2DM by modulating pancreatic β-cell dysfunction and insulin resistance in liver as a result from gut microbiota regulation.

## Author Contributions

ML, IT, and GL conceptualized the idea. ML, IT, and ST conducted the literature review and wrote the first draft of the manuscript. BL, RL, and PY assisted in the data collection and collation process. J-LC, YQ, CL, YL, MY, DY, GM, PR, XH, DX, and SC provided feedbacks and edited the manuscript. ML, LY, and PL provided suggestions in the treatment and diagnosis of diabetes to make this work more sensible. YH and GL provided many updated resources to enhance the quality of this work and contributed to the restructuring, editing process, and reviewing of the article. All authors agree with the final draft of the manuscript.

## Funding

Partial financial support was received from Sichuan Science and Technology Program (2019YFS0160).

## Conflict of Interest

DX, SC, and YH was employed by the company Chengdu Tepu Biotech Co. The remaining authors declare that the research was conducted in the absence of any commercial or financial relationships that could be construed as a potential conflict of interest. The authors declare that this study received funding from Sichuan Science and Technology Program. The funder was not involved in the study design, collection, analysis, interpretation of data, the writing of this article or the decision to submit it for publication.

## Publisher's Note

All claims expressed in this article are solely those of the authors and do not necessarily represent those of their affiliated organizations, or those of the publisher, the editors and the reviewers. Any product that may be evaluated in this article, or claim that may be made by its manufacturer, is not guaranteed or endorsed by the publisher.
