# Supplementary material for: Mechanisms and Active Compounds Polysaccharides and Bibenzyls of Medicinal Dendrobiums for Diabetes Management
Source: Front Nutr. 2022 Jan 28;8:811870. doi: 10.3389/fnut.2021.811870 (PMC8832146; doi:10.3389/fnut.2021.811870)
Supplement: Supplementary file 1 [file Table_1.docx]

Supplementary Material

# Supplementary Tables

## Supplementary Table 1. Anti-diabetic complication property of Dendrobium

| **Active compound** | ***Dendrobium* species** | **Model** | **Dosage** | **Parameters** | **Reference** |
| --- | --- | --- | --- | --- | --- |
| Polysaccharide | *D. huoshanense* | STZ induced of Sprague-Dawley rats | 50, 100 and 200 mg/kg, orally | Opacity of lenses ↓  Level of NO & NOS↓  iNOS expression ↓  AGEs production ↓ | (1) |
|  | *D. huoshanense* | BSA/glucose model:  Bovine serum albumin (2%)  Glucose 0.1 M | 0.1, 0.3 and 0.5 mg/mL | Inhibition of protein glycation in dose and time dependent manner | (2) |
|  | *D. huoshanense* | BSA/glucose model:  Bovine serum albumin 20 mmol/mL  Glucose 500mM | 0.1 and 1 mg/mL | Inhibition of formation Amadori products  AGEs production ↓ | (3) |
|  | *D. huoshanense* | BSA/glucose model:  Bovine serum albumin 20 M  Glucose 500 mM | 0.1 and 1 mg/mL | Inhibition of formation Amadori products  AGEs production ↓ | (4) |
|  | *D. huoshanense* | STZ induced C57BL/6 mice | 50 and 200 mg/kg | Reduced the fasting blood glucose of diabetic mice  Increased HOMA-β and reduced HOMA-IR  Suppressed β-cell apoptosis of pancreatic islet  Induced the secretion of GLP-1  Upregulated the phosphorylation of IRS-1, PI3K, and Akt | {Wang, 2019 #282}(5) |
| Polysaccharide | *D. officinale* | STZ induced of Kunming mice | 75, 150 & 300 mg/kg | Activities of myocardial enzymes CK and LDH ↓ on medium and high doses  Level of malondialdehyde in serum↓  Level of T-SOD in serum ↑  Lipid accumulation on cardiac tissue ↓  Expression of TGF-β, collegan-1 and fibronectin ↓ on medium and high doses | (6) |
|  | *D. officinale* | STZ induced Wistar rats | 20, 40, 80, 160mg/kg | Increased SOD level  Reduced MDA concentration  Reduced the concentration of IL-6 and TNF-α  Increased the concentration of IL-10  Reduced the concentration of fatty acid and glycerophospholipids | (7) |
|  | *D. officinale* | STZ induced C57BL/6J mice | 100, 200, and 400 mg/kg | Suppressed fasting blood glucose level  Decreased serum glucagon level  Inhibited cAMP-PKA signaling pathway  Upregulated the Akt/FoxO1 signaling pathway | (8) |
| Gigantol | *D. chrysotoxum* | High glucose induced on Human lens epithelial cells (HLECs) | 1 µg/mL | Expression of Aldose reductase gene ↓ | (9) |
| Erianin | *D. chrysotoxum* | High glucose induced on RF/6A cell | 1, 5, 10, 25 and 50 nM | No cytotoxicity effects  Expression of VEGF mRNA ↓  Translocation of HIF-1α into nucleus ↓  Inhibited VEGF-induced ERK1/2 phosphorylation and its downstream c-Raf and MEK1/2  Inhibited VEGF-induced PI3K phosphorylation and its downstream AKT, mTOR and P70S6 kinase | (10) |
|  | *D. chrysotoxum* | STZ induce C57BL/6 mice | 1 and 10 mg/kg | The increased of retinal vessel on Ganglion cell layer, inner & outer nuclear layer ↓  The increased of VEGF content in serum and vitreous cavity ↓  The increased of mRNA expression of VEGF in retinas↓  No effect on blood glucose concentration | (10) |
|  | *D. chrysotoxum* | STZ induce C57BL/6 mice | 1 and 10 mg/kg | Reduced the elevated retinal vascular permeability  Decreased the expression of occludin and claudin1.  Inhibited the activity microglial cell by reduced Iba1 expression.  Reduced the elevated p65 and p-p65  Decreased ERK1/2 phosphorylation. | (11) |
|  | *D. chrysotoxum* | High glucose induced on NRK-52E cell | 5, 10, 25, 50nM | Decreased LDH  Reduced ROS and MDA level  Inhibited release of cytocrome c  Decreased the expression of caspase 3 and 9  Downregulated the phosphorylation of p53 | (12) |
| Crude extract | *D. chrysotoxum* | STZ induced Sprague-Dawley rats | 30, 100 & 300 mg/kg | - no effect on serum glucose level  - CD31 (PECAM-1) ↓  - The increase of retinal vessel on Ganglion cell layer, inner & outer nuclear layer ↓  - expression of VEGF & VEGFR2 ↓  - serum level IL-6 and IL-1β ↓  - serum level and mRNA expression of MMP2 ↓ (100 & 300mg/kgBW)  - serum level and mRNA expression of MMP9 ↓(300mg/kgBW) | (13) |
|  | *D. chrysotoxum* | STZ induced Sprague-Dawley rats | 30 & 300 mg/kg | - no effect on serum glucose level  - mRNA expression of ICAM-1, TNF-α, IL-6, IL-1β ↓  - serum levels of TNF-α, IFN-λ, IL-6, IL-1β, IL-8, IL-12, IL-2, IL-10 ↓ | (14) |
|  | *D. candidum* | STZ-induced rats | 200, 400 and 800 mg/kg | Decreased the level of Src, BUN and urine protein  Downregulated VEGF expression  Decreased the expression of GLUT-1 and CTGF | (15) |
|  | *D. officinale* | STZ induced Sprague-Dawley rats | 10, 20g/kg | Reduced fasting blood glucose and fasting insulin  No significant effect on liver function  Reduced the expression of TLR2, TLR4, MyD88  Reduced HOMA-IR, FINS and GLU  Reduced TNF-αand IL-6 | (16) |

(1)

## Supplementary Table 2. Anti-inflammatory property of Dendrobium

| **Active compound** | ***Dendrobium* species** | **Model** | **Dosage** | **Parameters** | **Reference** |
| --- | --- | --- | --- | --- | --- |
| Polysaccharide | *D. chrysotoxum* | Mouse Splenocyte: cytotoxic assay | 50, 100 and 200 µg/mL | No cytotoxic effect, even at dose 200 µg/mL | (17) |
|  | *D. chrysotoxum* | Mouse Splenocyte: cytotoxic assay | 40 µg/mL | - Level of IFNγ and IL-6 ↑ - No change on IL-4 level | (17) |
| Phenanthrene  (12 different compounds) | *Dendrobium denneanum* | RAW264.7 cell:  cytotoxic assay LPS induced | 1, 50 and 100 µM | - no cytotoxic effect - inhibited NO production (potent inhibitory activities with IC_50_ values of 0.7-7.6 µM) - inhibited iNOS expression - inhibited p38, JNK (c-Jun N-terminal Kinase) MAPK and IκBα phosphorylation | (18) |
| Phenanthrene  - ephemeranthol (EA)  - 1,5,7-Trimethoxyphenanthren-2-ol (TP  - Dehydroorchinol (DO) | *D. nobile* | RAW264.7 cell:  cytotoxic assay LPS induced | 6.25 – 50 µg/mL | - only showed cytotoxic effect at concentration 50 µg/mL - EA and DO inhibited NO production but not TP - Inhibition of iNOS expression by EA and DO - EA and DO reduced COX-2 production - Level of TNF-α, IL-6 and IL-1β were decreased - EA and DO inhibited p38, JNK and ERK phosphorylation | (19) |
| Phenanthrene  - denbinobin | *D. moniliforme* | RAW264.7 cell: | 1 µM | - Inhibited TNFα and PGE_2_ | (20) |
| Phenanthrene  - dendrochrysanene | *D. chrysanthum* | Peritoneal macrophages C57BL/6J mice | 11.2 µg/mL | - Inhibited mRNAs level of TNFα, IL-8, IL-10 and iNOS | (21) |
| Alkaloid | *D. nobile* | LPS induce Sprague-Dawley rats | 40, 80 & 160 mg/kg | - expressions of TNFR1, NF-кB, and p38 MAPK of the hippocampus ↓ | (22) |
| Aqueous extract | *D. candidum* | CCl_4_ induced ICR mice | 200 &400 mg/kg | - serum level of AST, ALT LDH ↓ (400 mg/kg)  - degree of hepatic damage ↓  - serum levels of TNF-α, IFN-γ, IL-6, IL-12↓   - expression of NF-κB, COX-2, iNOS ↓ | (23) |

# References

1. Luo J-P, Deng Y-Y, Zha X-Q. Mechanism of Polysaccharides from Dendrobium huoshanense. on Streptozotocin-Induced Diabetic Cataract. *Pharm Biol* (2008) 46(4):243-9. doi: 10.1080/13880200701739397.

2. Pan LH, Feng BJ, Wang JH, Zha XQ, Luo JP. Structural characterization and anti-glycation activity in vitro of a water-soluble polysaccharide from dendrobium huoshanense. *J Food Biochem* (2013) 37. doi: 10.1111/j.1745-4514.2011.00633.x.

3. Qian X-P, Zha X-Q, Xiao J-J, Zhang H-L, Pan L-H, Luo J-P. Sulfated modification can enhance antiglycation abilities of polysaccharides from Dendrobium huoshanense. *Carbohydrate Polymers* (2014) 101:982-9. doi: <http://dx.doi.org/10.1016/j.carbpol.2013.10.035>.

4. Pan LH, Li XF, Wang MN, Zha XQ, Yang XF, Liu ZJ, et al. Comparison of hypoglycemic and antioxidative effects of polysaccharides from four different Dendrobium species. *Int J Biol Macromol* (2014) 64:420-7. Epub 2013/12/29. doi: 10.1016/j.ijbiomac.2013.12.024. PubMed PMID: 24370475.

5. Wang H-Y, Li Q-M, Yu N-J, Chen W-D, Zha X-Q, Wu D-L, et al. Dendrobium huoshanense polysaccharide regulates hepatic glucose homeostasis and pancreatic β-cell function in type 2 diabetic mice. *Carbohydrate Polymers* (2019) 211:39-48. doi: <https://doi.org/10.1016/j.carbpol.2019.01.101>.

6. Zhang Z, Zhang D, Dou M, Li Z, Zhang J, Zhao X. Dendrobium officinale Kimura et Migo attenuates diabetic cardiomyopathy through inhibiting oxidative stress, inflammation and fibrosis in streptozotocin-induced mice. *Biomed Pharmacother* (2016) 84:1350-8. Epub 2016/11/03. doi: 10.1016/j.biopha.2016.10.074. PubMed PMID: 27802903.

7. Yang J, Chen H, Nie Q, Huang X, Nie S. Dendrobium officinale polysaccharide ameliorates the liver metabolism disorders of type II diabetic rats. *Int J Biol Macromol* (2020) 164:1939-48. Epub 2020/08/09. doi: 10.1016/j.ijbiomac.2020.08.007. PubMed PMID: 32763406.

8. Liu Y, Yang L, Zhang Y, Liu X, Wu Z, Gilbert RG, et al. Dendrobium officinale polysaccharide ameliorates diabetic hepatic glucose metabolism via glucagon-mediated signaling pathways and modifying liver-glycogen structure. *J Ethnopharmacol* (2020) 248:112308. Epub 2019/10/18. doi: 10.1016/j.jep.2019.112308. PubMed PMID: 31622745.

9. Wu J, Li X, Wan W, Yang Q, Ma W, Chen D, et al. Gigantol from Dendrobium chrysotoxum Lindl. binds and inhibits aldose reductase gene to exert its anti-cataract activity: An in vitro mechanistic study. *Journal of Ethnopharmacology* (2017) 198:255-61. doi: <https://doi.org/10.1016/j.jep.2017.01.026>.

10. Yu Z, Zhang T, Gong C, Sheng Y, Lu B, Zhou L, et al. Erianin inhibits high glucose-induced retinal angiogenesis via blocking ERK1/2-regulated HIF-1alpha-VEGF/VEGFR2 signaling pathway. *Sci Rep* (2016) 6:34306. Epub 2016/09/30. doi: 10.1038/srep34306. PubMed PMID: 27678303; PubMed Central PMCID: PMCPMC5039671.

11. Zhang T, Ouyang H, Mei X, Lu B, Yu Z, Chen K, et al. Erianin alleviates diabetic retinopathy by reducing retinal inflammation initiated by microglial cells via inhibiting hyperglycemia-mediated ERK1/2-NF-kappaB signaling pathway. *FASEB J* (2019) 33(11):11776-90. Epub 2019/08/01. doi: 10.1096/fj.201802614RRR. PubMed PMID: 31365278; PubMed Central PMCID: PMCPMC6902687.

12. Chen M-F, Liou S-S, Kao S-T, Liu IM. Erianin protects against high glucose-induced oxidative injury in renal tubular epithelial cells. *Food and Chemical Toxicology* (2019) 126:97-105. doi: <https://doi.org/10.1016/j.fct.2019.02.021>.

13. Yang S, Gong Q, Wu Q, Li F, Lu Y, Shi J. Alkaloids enriched extract from Dendrobium nobile Lindl. attenuates tau protein hyperphosphorylation and apoptosis induced by lipopolysaccharide in rat brain. *Phytomedicine* (2014) 21(5):712-6. doi: <http://dx.doi.org/10.1016/j.phymed.2013.10.026>.

14. Yu Z, Gong C, Lu B, Yang L, Sheng Y, Ji L, et al. Dendrobium chrysotoxum Lindl. alleviates diabetic retinopathy by preventing retinal inflammation and tight junction protein decrease. *J Diabetes Res* (2015) 2015:518317. Epub 2015/02/17. doi: 10.1155/2015/518317. PubMed PMID: 25685822; PubMed Central PMCID: PMCPMC4313057.

15. Chang J, Zhou Y, Cong G, Guo H, Guo Y, Lu K, et al. Dendrobium candidum protects against diabetic kidney lesions through regulating vascular endothelial growth factor, Glucose Transporter 1, and connective tissue growth factor expression in rats. *Journal of Cellular Biochemistry* (2019) 120(8):13924-31. doi: <https://doi.org/10.1002/jcb.28666>.

16. Zhao M, Han J. Dendrobium Officinale Kimura et Migo Ameliorates Insulin Resistance in Rats with Diabetic Nephropathy. *Medical science monitor basic research* (2018) 24:84-92. doi: 10.12659/MSMBR.909242. PubMed PMID: 29849017.

17. Zhao Y, Son YO, Kim SS, Jang YS, Lee JC. Antioxidant and anti-hyperglycemic activity of polysaccharide isolated from Dendrobium chrysotoxum Lindl. *J Biochem Mol Biol* (2007) 40(5):670-7. Epub 2007/10/12. doi: 10.5483/bmbrep.2007.40.5.670. PubMed PMID: 17927899.

18. Lin Y, Wang F, Yang L-j, Chun Z, Bao J-k, Zhang G-l. Anti-inflammatory phenanthrene derivatives from stems of Dendrobium denneanum. *Phytochemistry* (2013) 95:242-51. doi: <http://dx.doi.org/10.1016/j.phytochem.2013.08.008>.

19. Kim JH, Oh SY, Han SB, Uddin GM, Kim CY, Lee JK. Anti-inflammatory effects of Dendrobium nobile derived phenanthrenes in LPS-stimulated murine macrophages. *Arch Pharm Res* (2015) 38(6):1117-26. Epub 2014/11/06. doi: 10.1007/s12272-014-0511-5. PubMed PMID: 25370607.

20. Lin TH, Chang SJ, Chen CC, Wang JP, Tsao LT. Two phenanthraquinones from Dendrobium moniliforme. *J Nat Prod* (2001) 64(8):1084-6. Epub 2001/08/25. doi: 10.1021/np010016i. PubMed PMID: 11520233.

21. Yang L, Qin LH, Bligh SW, Bashall A, Zhang CF, Zhang M, et al. A new phenanthrene with a spirolactone from Dendrobium chrysanthum and its anti-inflammatory activities. *Bioorg Med Chem* (2006) 14(10):3496-501. Epub 2006/01/25. doi: 10.1016/j.bmc.2006.01.004. PubMed PMID: 16431116.

22. Li Y, Li F, Gong Q, Wu Q, Shi J. Inhibitory effects of Dendrobium alkaloids on memory impairment induced by lipopolysaccharide in rats. *Planta Med* (2011) 77(2):117-21. Epub 2010/08/19. doi: 10.1055/s-0030-1250235. PubMed PMID: 20717874.

23. Gong C-Y, Yu Z-Y, Lu B, Yang L, Sheng Y-C, Fan Y-M, et al. Ethanol extract of Dendrobium chrysotoxum Lindl ameliorates diabetic retinopathy and its mechanism. *Vasc Pharmacol* (2014) 62(3):134-42. doi: <http://dx.doi.org/10.1016/j.vph.2014.04.007>.
